# Supplementary material for: Secreted Secondary Metabolites Reduce Bacterial Wilt Severity of Tomato in Bacterial–Fungal Co-Infections
Source: Microorganisms. 2021 Oct 9;9(10):2123. doi: 10.3390/microorganisms9102123 (PMC8537627; doi:10.3390/microorganisms9102123)
Supplement: Supplementary file 1 [file microorganisms-09-02123-s001.zip › microorganisms-1387089-supplementary.pdf]

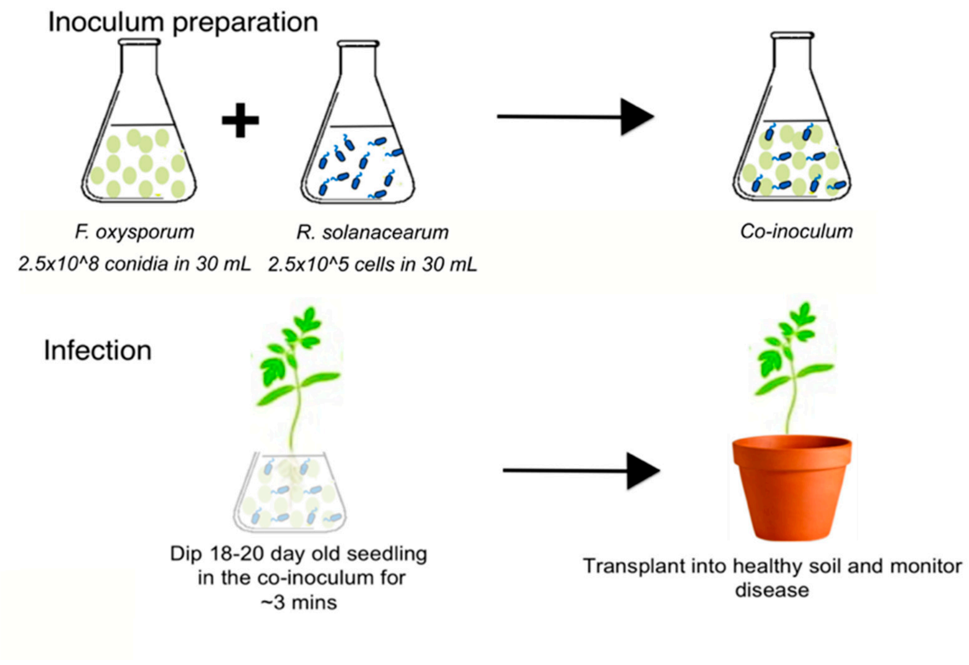

Figure S1. Schematic for co-infection of tomato seedlings. *Fusarium oxysporum* conidia are raised in PDB for 6 days at 30 °C with constant shaking at 250 rpm, post inoculation with two plugs of the fungus grown on PDA for 5 days. *R. solanacearum* is raised as overnight cultures by inoculating a single colony into 4 mL CPG broth at 30 °C with constant shaking at 250 rpm. The bacterial cells are harvested at OD>1.0.

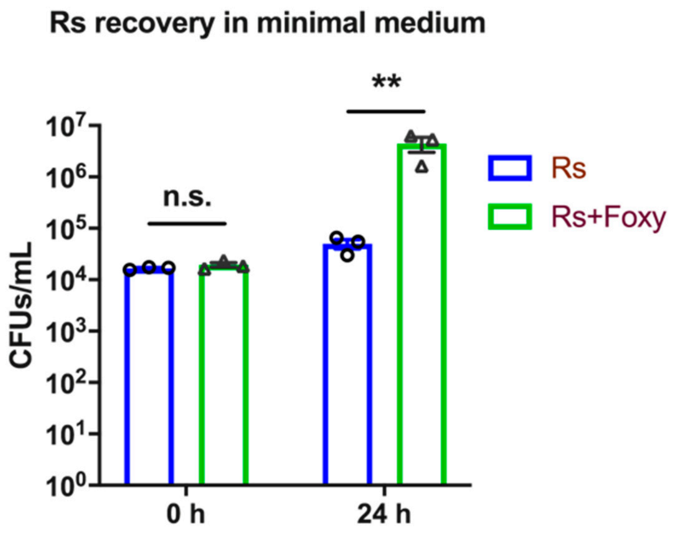

t-test

	P value
0 h	0.261072
24 h	0.037425

Figure S2. *R. solanacearum* growth is not inhibited in *in vitro* co-cultures. The figure shows colony forming units (CFUs) of *R. solanacearum* in single (Rs) and co-cultures with *F. oxysporum* *(*Rs+Foxy). Independent t-test was performed for each time point and the p-values are reported.

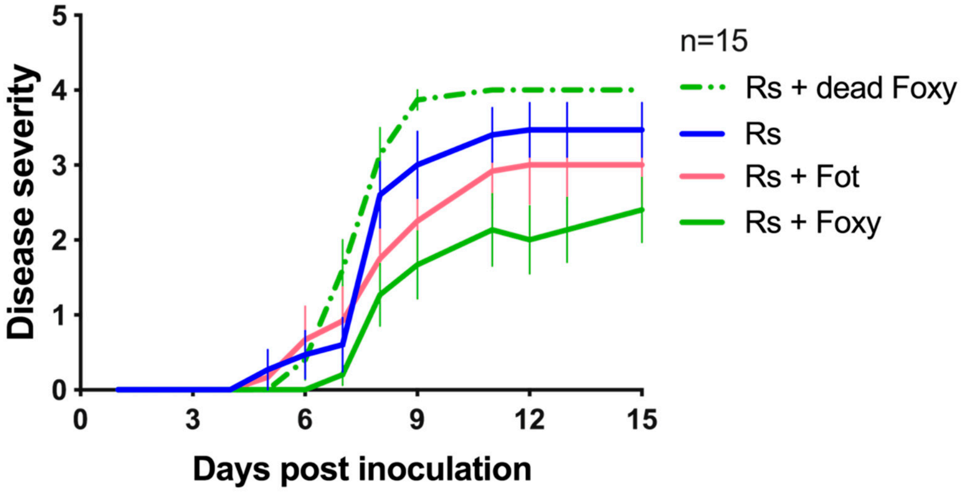

Two-way ANOVA Treatment x time: **p=0.0005

Tukey's multiple comparisons test	Summary	Adjusted P Value
Rs vs. Rs + Foxy	****	<0.0001
Rs vs. Rs + Fot	ns	0.2136
Rs vs. Rs + dead Foxy	*	0.0227
Rs + Foxy vs. Rs + Fot	**	0.0013
Rs + Foxy vs. Rs + dead Foxy	****	<0.0001
Rs + Fot vs. Rs + dead Foxy	****	<0.0001

Figure S3. Reduction in bacterial wilt during co-infection requires active and specific interactions with *F. oxysporum f. sp. lycopersici.* Disease severity is calculated based on scores assigned daily. Rs + dead Foxy refers to co-infections of tomato plants with *R. solanacearum* and heat-killed *F. oxusporum* spores (60 °C for 8 hours). Rs + Fot refers to co-infection of tomato plants with *R. solanacearum* and *F. oxysporum f. sp. tulipae* which cannot infect tomato. All statistical analyses and p-values are listed in the figure.

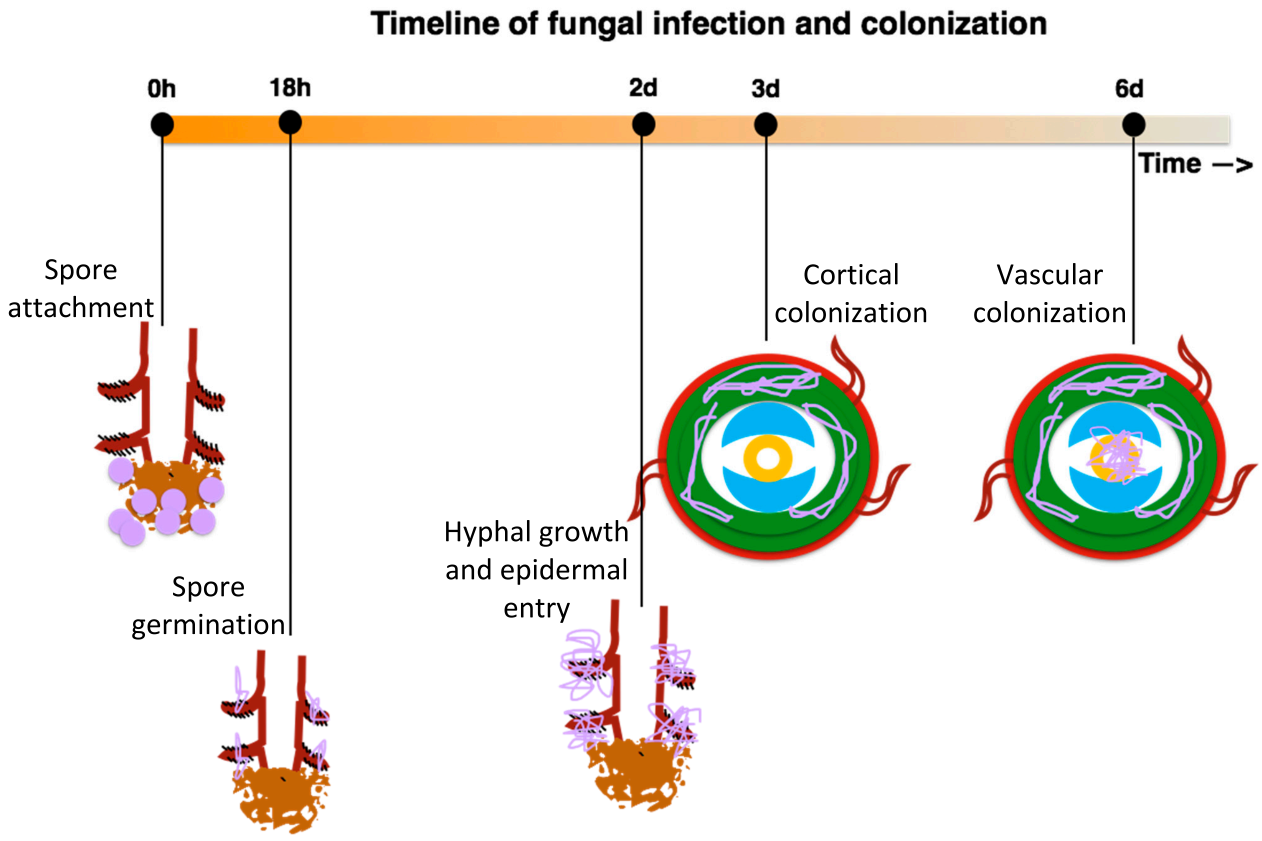

Figure S4. A timeline for *F. oxysporum* infection. The infection timeline begins from recognition of the host until colonization of the xylem, gathered from available literature. References used here include Olivain et al. 2006 [1] and Lagopodi et al. 2002 [2].

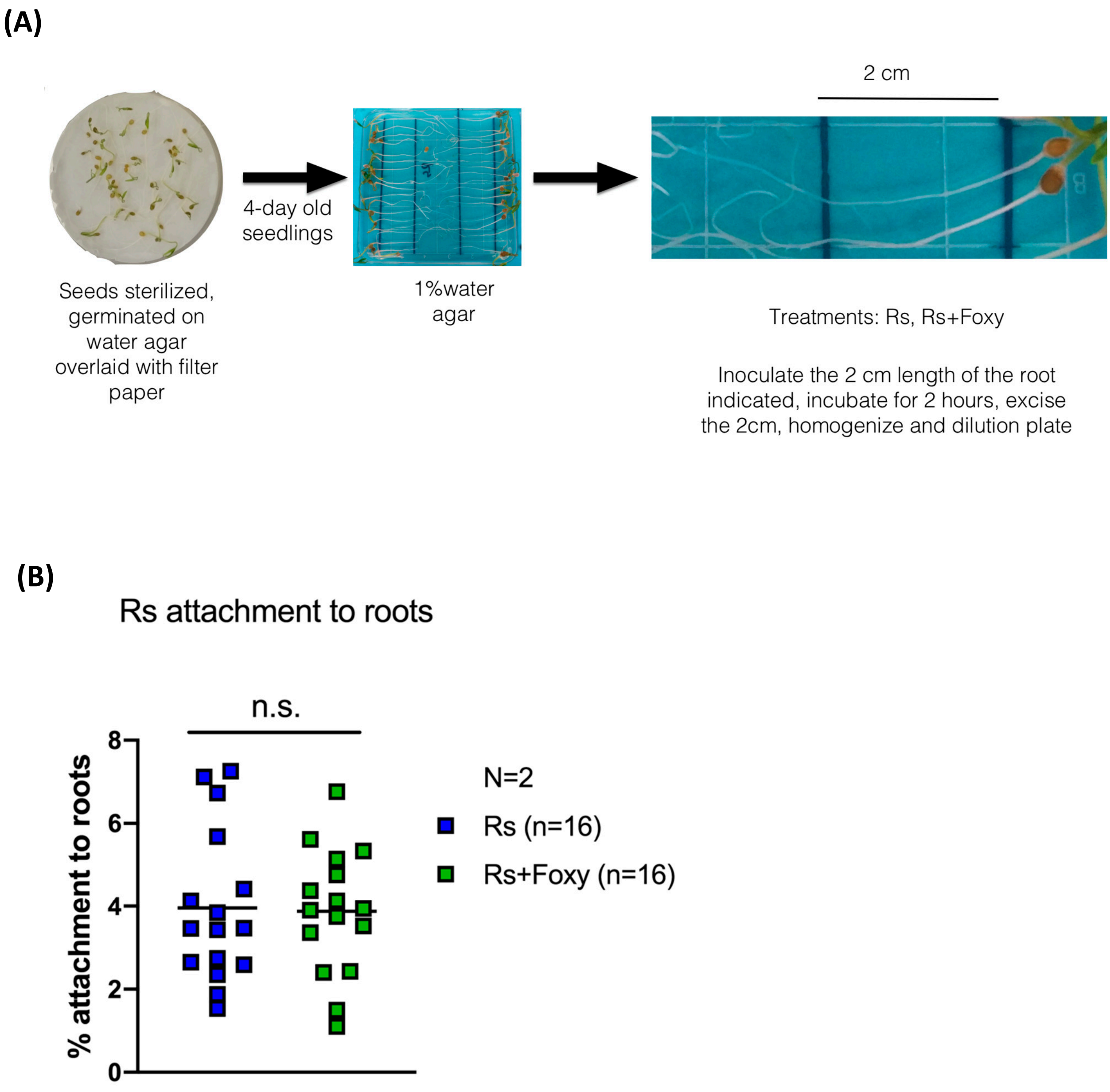

Unpaired t test with Welch's correction	
P value	0.8968

Figure S5. Assessment of *R. solanacearum* attachment to roots. (A) Schematic for quantification of *R. solanacearum* attachment to roots. (B) CFUs of *R. solanacearum* obtained from single infections (Rs) and co-infections (Rs+Foxy). The normality of the data was tested with the Shapiro-Wilt normality test.

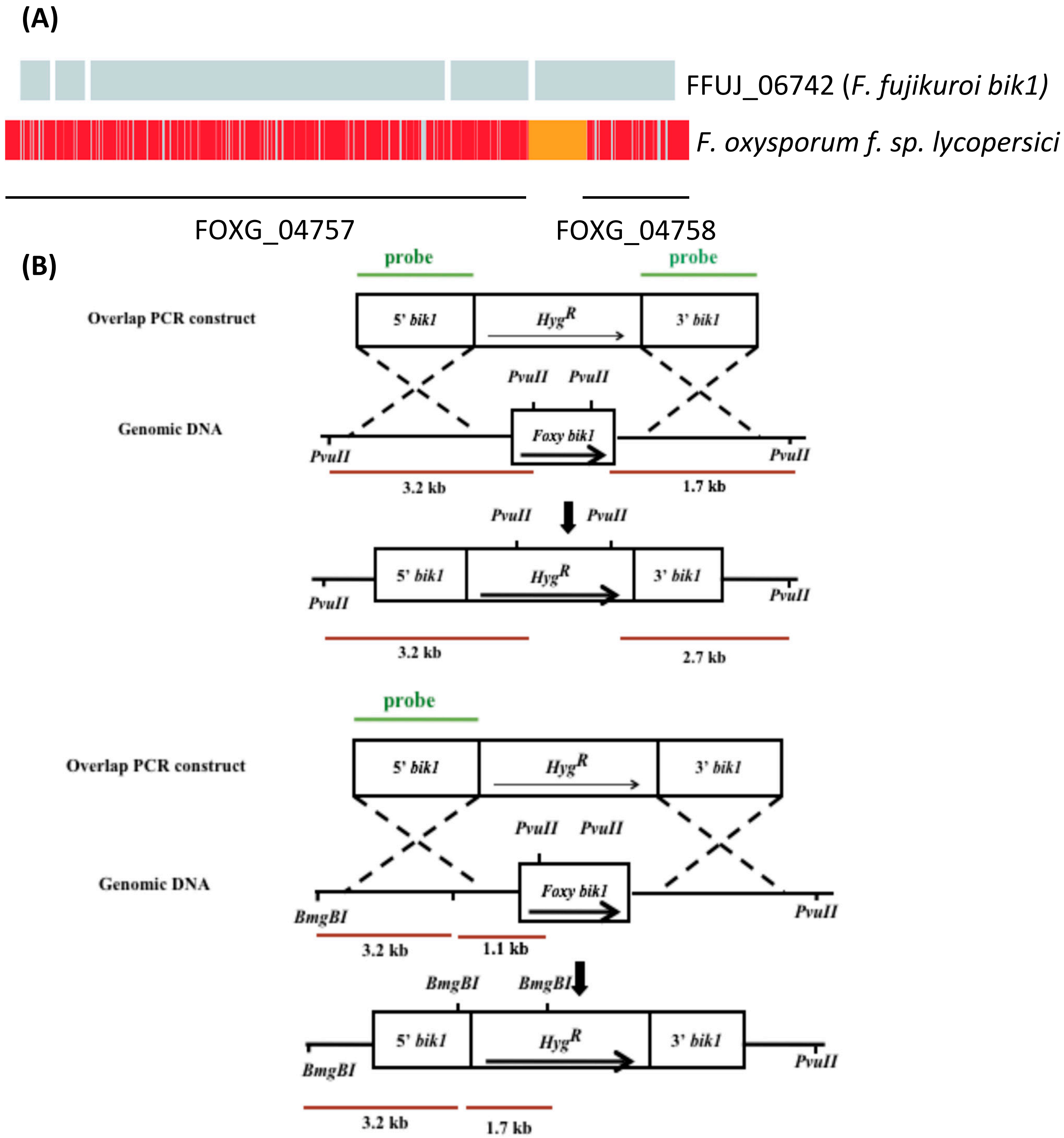
(A)
FFUJ_06742 (F. fujikuroi bik1)
F. oxysporum f. sp. lycopersici
FOXG_04757
FOXG_04758
(B)
probe
probe
Overlap PCR construct
5' bik1
HygR
3' bik1
PvuII
PvuII
Genomic DNA
Foxy bik1
PvuII
PvuII
3.2 kb
1.7 kb
PvuII
PvuII
5' bik1
HygR
3' bik1
PvuII
PvuII
3.2 kb
2.7 kb
probe
Overlap PCR construct
5' bik1
HygR
3' bik1
PvuII
PvuII
Genomic DNA
Foxy bik1
BmgBI
PvuII
3.2 kb
1.1 kb
BmgBI
BmgBI
5' bik1
HygR
3' bik1
BmgBI
PvuII
3.2 kb
1.7 kb

(C)

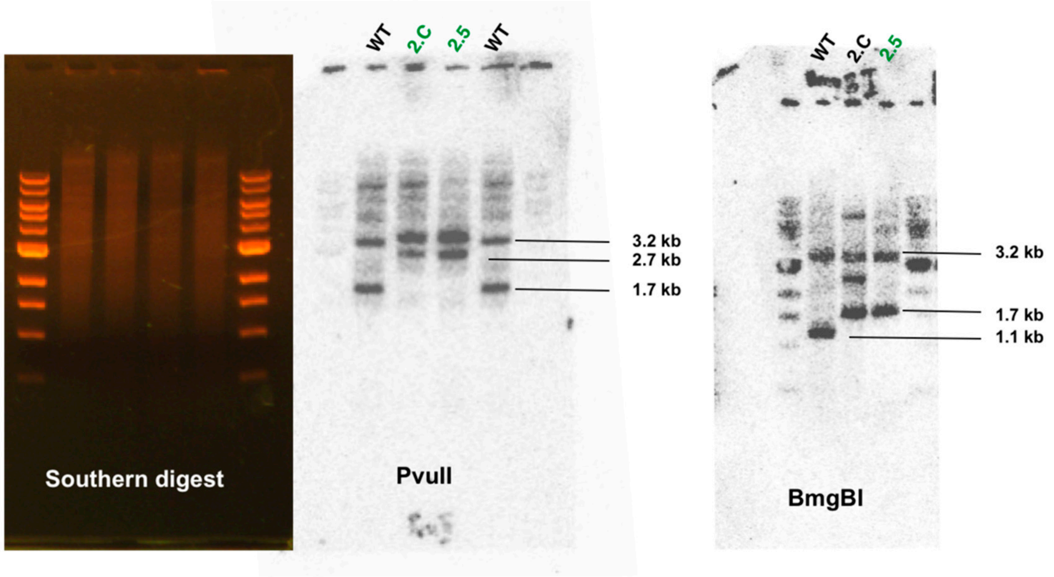

(D)

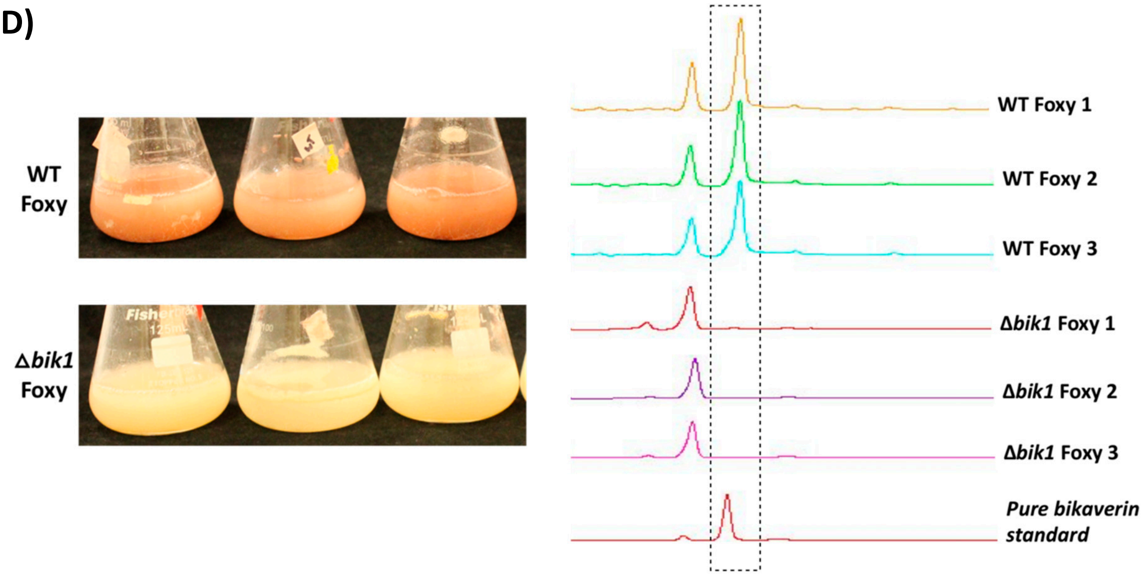

Figure S6. *bik1* knockout construction and validation. (A) Genomic context for *bik1* gene in *F. oxysporum*. The figure is a representation of sequence alignment between the *bik1* in *F. fujikuroi* (grey) and predicted orthologs in *F. oxysporum* identified based on protein and nucleotide blast (red). The orange region represents insufficient sequence information. (B) Schematic representation of the genetic construct used for *bik1* deletion in *F. oxysporum.* The construct was made using double fusion PCR and includes the *hph* gene that confers resistance to hygromycin (denoted in the figure as HygR). The flanking regions upstream and downstream of the *bik1* gene are denoted as 5’ and 3’ flanks and these enable homologous recombination. The restriction enzyme cut sites are indicated. (C) shows southern blot analyses of genomic DNA from the WT and Δ*bik1* strains (2.C and 2.5). Ten micrograms of total DNA from each strain was digested with

the enzymes of interest and subjected to southern blot analysis with 5’ flank and 3’ flank fragments as probes (indicated in A). The size of the bands was identified with the New England Biolabs 1 kb DNA ladder. Strain 2.5 was chosen to be used as the Δ*bik1* strain for future analyses. (D) Bikaverin production was analyzed from WT and Δ*bik1 F. oxysporum* strains. The strains were grown on PDA for 5 days at 30 °C and two plugs were transferred to 50 mL of liquid ICI media in 125 mL flasks. These were incubated at 30 °C for 1 week. The cultures were extracted with ethyl acetate and the crude extract was analyzed with HPLC. Pure bikaverin at 0.1 mg/mL was used as the standard.

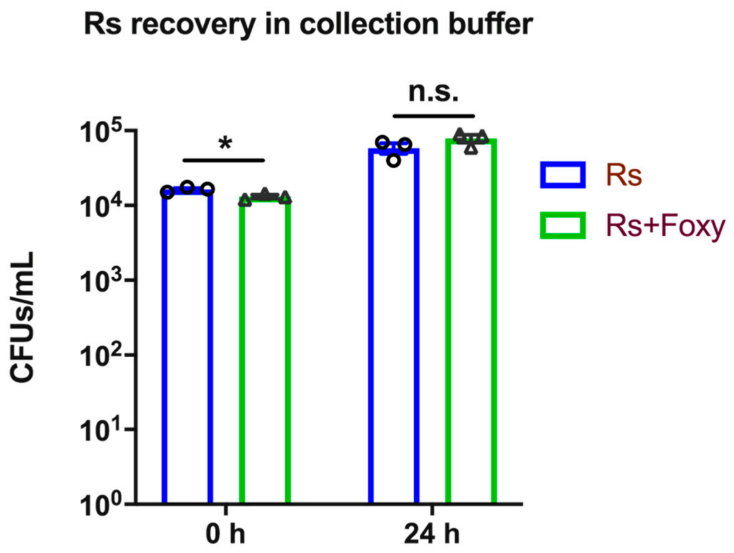

	Significant?	P value
0 h	Yes	0.036852397146530
24 h	No	0.202185643384270

Figure S7. Co-culture of *R. solanacearum* and *F. oxysporum* in secretome collection buffer does not inhibit bacterial growth. CFUs of the bacterium in single and co-cultures in the collection buffer used to obtain early infection secretome. 24 hours was chosen as the secretome is collect after a 24-hour incubation.

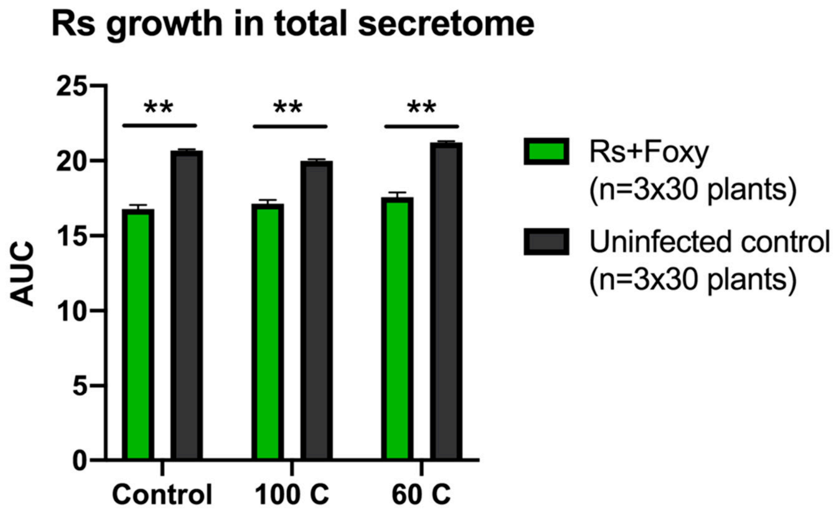

t-test

	Significant?	P value
Control	Yes	<0.000001
100 C	Yes	<0.000001
60 C	Yes	<0.000001

Figure S8. The bioactive molecules in the co-infection secretome do not degrade with heat. Total secretomes from co-infected plants and uninfected control plants were heat treated at 100 °C (5 min) or 60 °C (2 hours) with room temperature control. *R. solanacearum* growth in the treated-secretomes were quantified with OD_{600} and area under the growth curve (AUC) was calculated.

(A)

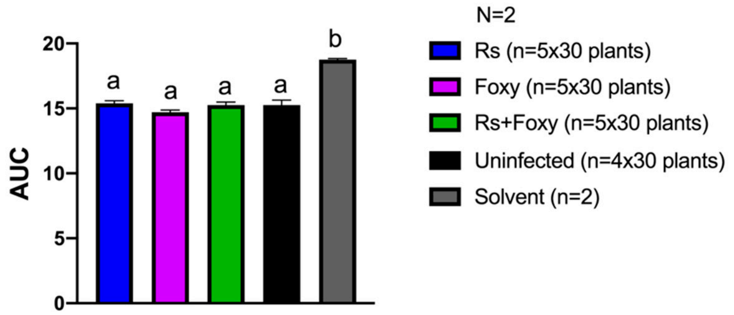

ANOVA **P<0.0001

Tukey's multiple comparisons test	Summary	Adjusted P Value
Rs vs. Foxy	ns	0.2918
Rs vs. Rs+Foxy	ns	0.9928
Rs vs. Uninfected	ns	0.9943
Rs vs. Solvent	****	<0.0001
Foxy vs. Rs+Foxy	ns	0.5062
Foxy vs. Uninfected	ns	0.5604
Foxy vs. Solvent	****	<0.0001
Rs+Foxy vs. Uninfected	ns	>0.9999
Rs+Foxy vs. Solvent	****	<0.0001
Uninfected vs. Solvent	****	<0.0001

(B)

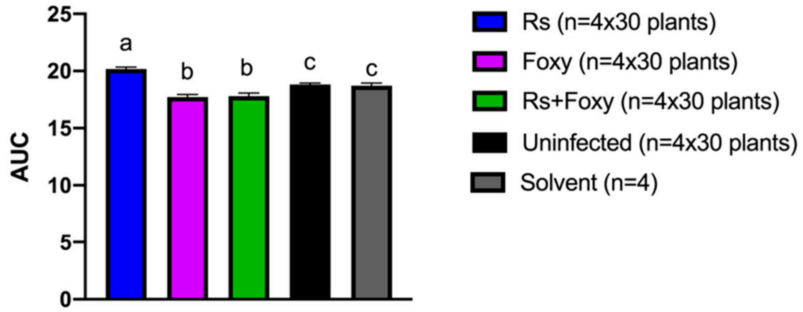

ANOVA **p<0.0001

Tukey's multiple comparisons test	Summary	Adjusted P Value
Rs vs. Foxy	****	<0.0001
Rs vs. Rs+Foxy	****	<0.0001
Rs vs. Uninfected	**	0.0041
Rs vs. Solvent	**	0.0019
Foxy vs. Rs+Foxy	ns	0.9982
Foxy vs. Uninfected	*	0.0167
Foxy vs. Solvent	*	0.0354
Rs+Foxy vs. Uninfected	*	0.0294
Rs+Foxy vs. Solvent	ns	0.0614
Uninfected vs. Solvent	ns	0.9944

Figure S9. *R. solanacearum* growth in secondary metabolite crude extracts eluted into hexane (A) and methanol (B). The crude extracts were extracted from total secretomes in different solvents as mentioned. *R. solanacearum* growth in the treated-secretomes were quantified with OD600 and area under the growth curve (AUC) was calculated.

(A)

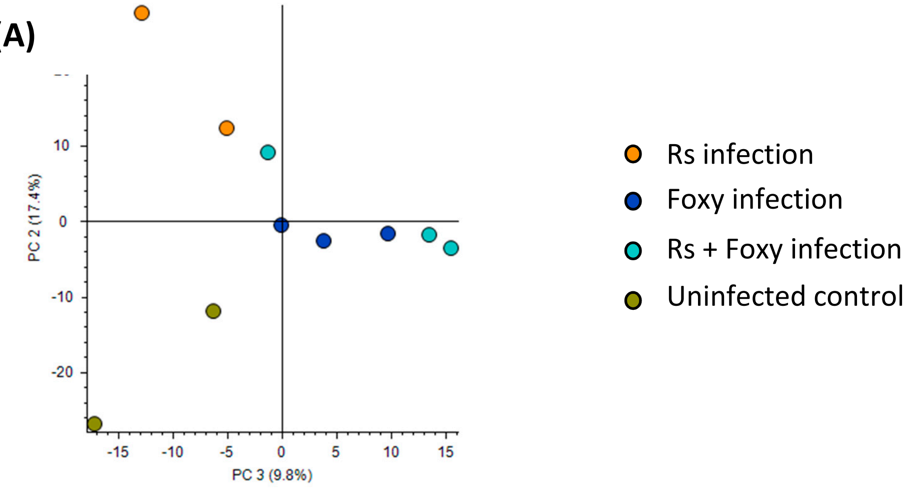

PC 2 (17.4%)
PC 3 (9.8%)
Rs infection
Foxy infection
Rs + Foxy infection
Uninfected control

(B)

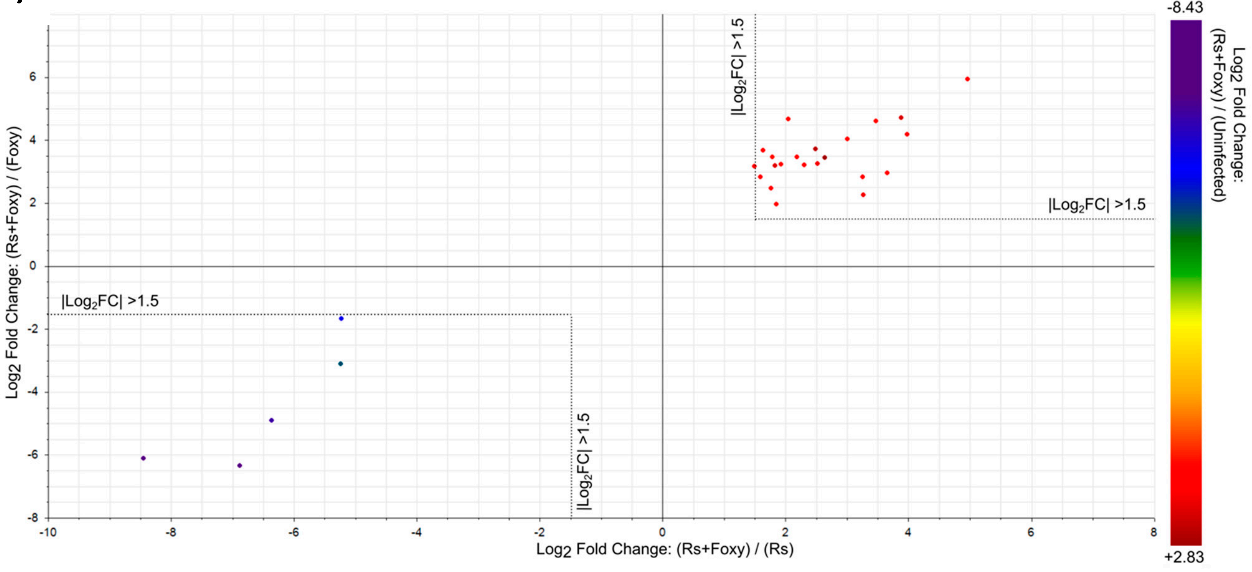

Log2 Fold Change: (Rs+Foxy) / (Foxy)
Log2 Fold Change: (Rs+Foxy) / (Rs)
|Log2FC| >1.5
|Log2FC| >1.5
|Log2FC| >1.5
|Log2FC| >1.5
Log2 Fold Change: (Rs+Foxy) / (Uninfected)
-8.43
+2.83

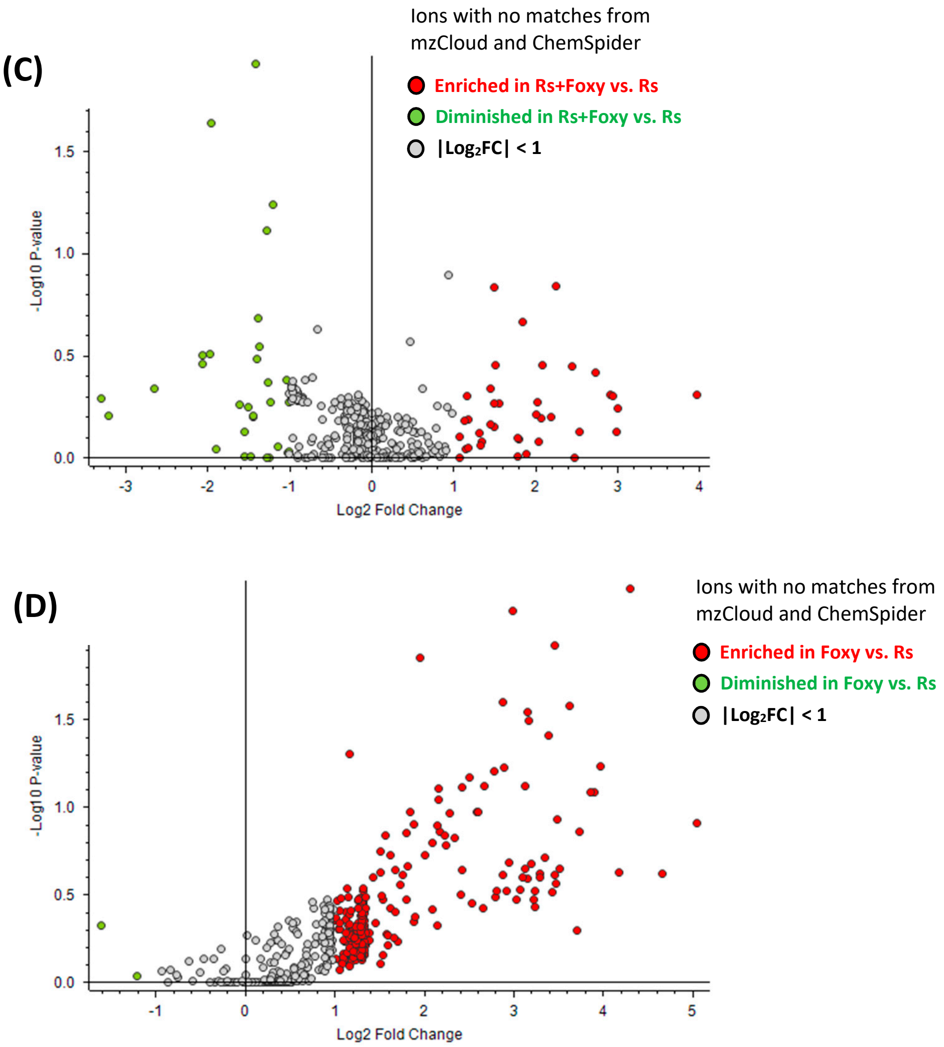

Figure S10. Untargeted metabolomic analyses of crude extracts from single and co-infected secretomes. (A) Ordination plot showing clustering of treatments. While 2/3 replicates of co-infection extract cluster together, one replicate lies between Rs and Foxy single-infections. (B) shows ions enriched (red) or diminished (purple/blue) exclusively in co-infections. Log2 Fold change of the ions in co-infection compared to Rs single infection, Foxy single infection and uninfected control is plotted on the x-axis, y-axis and z-axis color scale respectively. A minimum Log2 fold change of 1.5 is used as a threshold. Ions in the top right quadrant are enriched in co-infection compared to both Rs and Foxy single-infections and the uninfected control. Ions in the bottom left quadrant are diminished in co-infection compared to both Rs and Foxy single-infections and the uninfected control. (C) and (D) show ions that returned no matching annotations from mzCloud and ChemSpider. (C) shows ions enriched (red) or diminished (green) in Rs+Foxy co-infection compared to Rs single-infection. (D) shows ions enriched (red) or diminished (green) in Rs+Foxy co-infection compared to Foxy single-infection.

Sample	Peak	ES-	ES+	Formula	Formula		Known?
**10**	A	323.2335		C18H34O5	C19H30ON4		
			353.229	C16H28O3N6			
	B	667.2825		C30H44O13N4	C29H48O17	C31H40O9N8	
			640.3160	C28H49O15N			
	E	374.2448<br>420.2501	376.2588	C21H33O3N3<br>C22H35O5N3	C23H31ON7		Terestigmine?
**12**	C	681.2977		C30H50O17	C32H42O9N8		Salicinolide?
			654.3315	C38H39O2N9	C30H47N5O11		
	E			C21H33O3N3<br>C22H35O5N3	C23H31ON7		
**13**	D	681.2080		C31H46O13N4	C30H50O17	C32H42O9N8	
			654.3315	C38H39O2N9			
	E	374.2448<br>420.2501	376.2588	C21H33O3N3<br>C22H35O5N3	C23H31ON7		
**14**	E	374.2448<br>420.2501	376.2588	C21H33O3N3<br>C22H35O5N3	C23H31ON7		
**15**	E	374.2448<br>420.2501	376.2588	C21H33O3N3<br>C22H35O5N3	C23H31ON7		

Table S1. m/z values for compounds shown in the chromatograms from figure 4D and their formulas predicted by compound discoverer 3.2 and XCalibur software Version 3.1.66.10. The left-most column shows lists the fractions corresponding to Figure 4C,D and Figure 5.

**Fungal/bacterial strain**	**Genotype**	**Reference/Source**
Fusarium oxysporum f. sp. lycopersici 4287	Wild Type	Strain from Dr. Antonio Di Pietro, University of Córdoba, Spain. Di Pietro et al. 2004 [3]
Δ*bik1 Fusarium oxysporum f. sp. lycopersici* 4287	Δ*bik1*::Hyg^R (Wild Type as parent)	This study
Fusarium oxysporum f. sp. tulipae 26954	Wild Type	Strain from Dr. Ernst Oliw, Uppsala University, Sweden. Oliw et al. 2019 [4]
Ralstonia solanacearum GMI1000	Wild Type	Strain from Dr. Caitilyn Allen, Univ. of Wisconsin-Madison, USA. Boucher et al. 1987 [5]

Table S2. List of strains used in the study**.**

Primer Name	Primer (5’ to 3’)
NV FOXY57 5’F NESTFOR	ATGCGCCGTCTTCGTCAACA
NV FOXY57 5’F FOR	AGGGCCTGCAACTACTCTTG
NV FOXY57 5’F REV	TGGAGCTCCAATTCGCCCTATAGAGTGTTGCACCTGCATGATCCT
NV FOXY58 3’F FOR	TAGTGAGGGTTAATTGCGCGCTTGCTCCCCCGGGTAAACATAACACT
NV FOXY58 3’F NESTREV	AGGCAATCGAGACTACCGGT
NV FOXY58 3’F REV	ACGCATCTCGGAGAGAATGAC
NV HygB 5’F	TATAGGGCGAATTGGAGCTCCA
NV HygB 3’F	CAAGCGCGCAATTAACCCTCACTA
NV foxy BIK1-int FOR	GAGATGAGAACGCAGAAGGCC
NV foxy BIK1-int REV	GTGTTCTTGCGCTGGCCA
NV foxy GPD-int FOR	GCTGCCTCTCGATAAGTGGTG
NV foxy GPD-int REV	GACGTGAACTCCAGATGCTGG

Table S3. List of primers used in the study

References for supplementary material:

1. Olivain, C.; Humbert, C.; Nahalkova, J.; Fatehi, J.; L'Haridon, F.; Alabouvette, C. Colonization of tomato root by pathogenic and nonpathogenic Fusarium oxysporum strains inoculated together and separately into the soil. *Appl. Environ. Microbiol.* **2006**, *72*, 1523–1531, doi:10.1128/AEM.72.2.1523-1531.2006.
2. Lagopodi, A.L.; Ram, A.F.J.; Lamers, G.E.M.; Punt, P.J.; Van den Hondel, C.A.M.J.J.; Lugtenberg, B.J.J.; Bloemberg, G. V. Novel aspects of tomato root colonization and infection by Fusarium oxysporum f. sp. radicis-lycopersici Revealed by confocal laser scanning microscopic analysis using the green fluorescent protein as a marker. *Mol. Plant-Microbe Interact.* **2002**, *15*, 172–179, doi:10.1094/MPMI.2002.15.2.172.
3. Di Pietro, A.; García-Maceira, F.I.; Méglecz, E.; Roncero, M.I.G. A MAP kinase of the vascular wilt fungus Fusarium oxysporum is essential for root penetration and pathogenesis. *Mol. Microbiol.* **2004**, *39*, 1140–1152, doi:10.1111/j.1365-2958.2001.02307.x.
4. Oliw, E.H.; Hamberg, M. Biosynthesis of Jasmonates from Linoleic Acid by the Fungus Fusarium oxysporum. Evidence for a Novel Allene Oxide Cyclase. *Lipids* **2019**, *54*, 543–556, doi:10.1002/lipd.12180.
5. Boucher, C.A.; Van Gijsegem, F.; Barberis, P.A.; Arlat, M.; Zischek, C. Pseudomonas solanacearum genes controlling both pathogenicity on tomato and hypersensitivity on tobacco are clustered. *J. Bacteriol.* **1987**, *169*, 5626–5632, doi:10.1128/jb.169.12.5626-5632.1987.
